# Haplotype Analyses of DNA Repair Gene Polymorphisms and Their Role in Ulcerative Colitis

**DOI:** 10.1371/journal.pone.0108562

**Published:** 2014-09-23

**Authors:** Avinash Bardia, Santosh K. Tiwari, Sandeep K. Vishwakarma, Md. Aejaz Habeeb, Pratibha Nallari, Shaik A. Sultana, Shaik A. Pasha, Yugandhar P. Reddy, Aleem A. Khan

**Affiliations:** 1 Centre for Liver Research and Diagnostics, Deccan College of Medical Sciences, Kanchanbagh, Hyderabad, Andhra Pradesh, India; 2 Department of Genetics, Osmania University, Hyderabad, Andhra Pradesh, India; 3 College of Applied Medical Sciences, King Saud University, Riyadh, Kingdom of Saudi Arabia; 4 Neurobiology lab, Department of Zoology, University College of Sciences, Osmania University, Hyderabad, Andhra Pradesh, India; University of Navarra, Spain

## Abstract

Ulcerative colitis (UC) is a major clinical form of inflammatory bowel disease. UC is characterized by mucosal inflammation limited to the colon, always involving the rectum and a variable extent of the more proximal colon in a continuous manner. Genetic variations in DNA repair genes may influence the extent of repair functions, DNA damage, and thus the manifestations of UC. This study thus evaluated the role of polymorphisms of the genes involved in DNA repair mechanisms. A total of 171 patients and 213 controls were included. Genotyping was carried out by ARMS PCR and PCR-RFLP analyses for *RAD51*, *XRCC*3 and *hMSH2* gene polymorphisms. Allelic and genotypic frequencies were computed in both control & patient groups and data was analyzed using appropriate statistical tests. The frequency of ‘A’ allele of *hMSH*2 in the UC group caused statistically significant increased risk for UC compared to controls (OR 1.64, 95% CI 1.16–2.31, *p* = 0.004). Similarly, the CT genotype of *XRCC*3 gene was predominant in the UC group and increased the risk for UC by 1.75 fold compared to controls (OR 1.75, 95% CI 1.15–2.67, *p* = 0.03), further confirming the risk of ‘T’ allele in UC. The GC genotype frequency of *RAD*51 gene was significantly increased (*p* = 0.02) in the UC group (50.3%) compared to controls (38%). The GC genotype significantly increased the risk for UC compared to GG genotype by 1.73 fold (OR 1.73, 95% CI 1.14–2.62, *p* = 0.02) confirming the strong association of ‘C’ allele with UC. Among the controls, the SNP loci combination of *hMSH*2:*XRCC*3 were in perfect linkage. The GTC and ACC haplotypes were found to be predominant in UC than controls with a 2.28 and 2.93 fold significant increase risk of UC.

## Introduction

The repair of damaged DNA forms an integral part of cell rejuvenation and is known to protect against different diseases [1]. Approximately four major DNA repair pathways viz., nucleotide excision repair (NER), base excision repair (BER), double strand break repair (DBR) and mismatch repair (MMR) [3]–[5] operate on specific types of damaged DNA [2]. A deficiency in DNA repair capacity due to gene mutations can lead to genomic instability and disease susceptibility [6].

The mismatch repair human mutS homolog 2 (*hMSH2*) genes are integral components of the DNA mismatch repair pathway. The *hMSH2* gene is located on chromosome 2p21, an area initially identified as an important candidate region for genes involved in hereditary nonpolyposis colorectal cancer (HNPCC) [9]. *hMSH2* can form a heterodimer with one of the two other mismatch repair proteins, *hMSH6* or *hMSH3* [10]. An amino acid substitution at codon 322 (*Gly*322*Asp*) of *hMSH*2 may affect the heterodimer formation with other proteins. Other investigators have demonstrated that genotypes in this gene have an increased risk for colorectal cancer [9]. The *XRCC3* and *RAD*51 gene encodes protein involved in homologous recombinational repair (HRR) of double strand DNA [11]. The *XRCC*3 gene has a sequence variation in exon 7 (C18067T), which result in an amino acid substitution at codon 241 (*Thr*241*Met*). This substitution may affect its interaction with other proteins involved in DNA damage and repair [12]. Several study demonstrated that *XRCC*3 polymorphisms are implicated in breast cancer [13], lung cancer [14]. Among several polymorphisms in *RAD*51 gene, a functional SNP at position 135 in 5′ UTR, changing a guanine to cytosine, was reported. Recently, two important meta-analyses [15]–[16], covering tens of other studies and thousands of subjects, were unanimous to state that the variant allele of *RAD51* G135C may contribute to increased breast cancer susceptibility, which is in accordance with biological function study, which showed a more aggressive and poor prognosis phenotype [17].

UC is complex disease with a strong genetic component [7]. It is presumed to be a multifactorial interplay between host genetics and environmental factors, leading to an aberrant inflammatory response [8]. The present study explored the impact of polymorphisms in important DNA repair genes (*hMSH2*, *XRCC*3, *RAD*51) in UC. The role of polymorphisms in these genes and their relation in UC needs to be elucidated to understand the pathogenesis of UC.

## Materials and Methods

### Patients and study design

A total of 171 patients (104 males and 67 females) with UC and 213 (131 males and 82 females) healthy volunteers were included. Healthy controls were selected without any prior history of GI presentations, autoimmune diseases and infections. There was no history of malignancy in the control group or in the UC patient group. The patients were selected from the symptomatic subjects who underwent colonoscopy at the Department of Gastroenterology, Deccan College of Medical Sciences, Hyderabad, India. To avoid selection bias, extensive care was taken not to include any patient with concomitant chronic inflammatory diseases such as arthritis, upper GI disorder etc. All the patients were asked to provide maximum information about their symptoms, disease, duration, and history of any disease that could affect the study outcome. The study protocol was approved by the Institutional Ethics Committee, Deccan College of Medical Sciences, Hyderabad, India. All study participants were asked to provide their written and signed consent to be part of this study protocol.

### Genotyping

Five milliliter (5 mL) of peripheral venous blood was collected by venipuncture from all the subjects. DNA was isolated from 200 µL whole blood using a commercially available kit (Bioserve Biotechnology Pvt. Ltd., Hyderabad, India). Polymorphisms at *Thr*241*Met* in the *XRCC*3 gene were screened by polymerase chain reaction (PCR) with specific primers as described elsewhere [18]. Polymorphisms at Gly322Asp in the *hMSH*2 gene and G135C in the *RAD*51 gene were determined by PCR-restriction fragment length polymorphism as described previously [19]. The 252 bp PCR product was digested at 37°C for 1 hour with 5 U of the restriction enzyme *Hinf*I. The *Asp* allele was identified by a 70 and 182 bp fragments and the *Gln* allele as 252 bp. For *RAD*51 gene the 157 bp PCR product was digested at 37°C for 1 hour with 5 U of the restriction enzyme *MvaI*. G allele was identified as 157 bp and C allele was identified by 86 and 71 bp fragments.

### Statistical Analysis

The sample size was determined by using the openEPi statistics and 95% of confidence was used to detect the results with 90% of sample power. Odds ratios, with 95% confidence intervals were calculated to compare allele and genotype frequencies. The extent of linkage disequilibrium (LD) was expressed in terms of the maximum likelihood estimate of disequilibrium, D′. For each of the SNPs, the covariates such as age and gender were adjusted. A Bonferroni test was performed for multiple testing because this method is valid for equal and unequal sample sizes and it can be used to correct any set of P values for multiple comparisons. Statistical analysis was carried out using SNPstats software, available online (www.bioinfo.iconcologia.net/SNPstats) and Haploview software. For all tests, significance level was set as p<0.05.

## Results

### Distribution of allelic and genotypic frequency of G322A polymorphisms in *hMSH*2 gene

The frequency of **‘A’** allele was found to be predominant in UC group compared to controls (27% vs 18% respectively), with a 1.64 folds increased risk for UC (OR 1.64, 95% CI 1.16–2.31, *p* = 0.004) **(Table 1)**. Heterozygotes (GA) were found to be predominant in the UC group compared to controls (49.1%, 35.7% respectively, *p* = 0.004) with 1.81 folds increased risk for UC, which was statistically significant (OR 1.81, 95% CI 1.20–2.74, *p* = 0.004). Based on the dominant model, combination of GA+AA genotypes was also observed to be associated with high risk for UC (OR 1.87, 95% CI 1.24–2.82, *p* = 0.002). In recessive model no such variation was observed (compared with GG+GA) (OR 5.08, 95% CI 0.56–45.81, *p* = 0.1) **(Table 1)**. Whereas in the overdominant model, GA (Compared with GG+AA genotype) genotype found to be associated with a 1.74 folds increased risk for UC (OR 1.74, 95% CI 1.15–2.62, *p* = 0.007), further confirming the risk of **‘A’** allele in UC **(Table 2)**.

**Table 1:** Allele frequency distributions of *hMSH*2 *XRCC*3 *RAD*51 (G135C) in UC and healthy controls.

	Controls (213)		Patients (171)			
Allele	N	Freq	N	Freq	OR 95% CI	*p*- value
*hMSH*2						
**G**	348	0.82	250	0.73	0.60 (0.43–0.85)	0.004
**A**	78	0.18	92	0.27	**1.64 (1.16–2.31)**	0.004
*XRCC*3						
**C**	345	0.81	256	0.75	0.69 (0.49–0.98)	0.041
**T**	81	0.19	86	0.25	**1.43 (1.01–2.01**)	0.041
*RAD*51 (G135C)						
**G**	335	0.79	242	0.71	0.65 (0.47–0.91)	0.012
**C**	91	0.21	100	0.29	**1.52 (1.09–2.11)**	0.012

**Table 2:** *hMSH*2 genotypic distribution in UC compared to healthy controls.

	Genotype	Controls	Patients	OR (95% CI)	*p*-value
**Co-dominant**	G/G	136 (63.9%)	83 (48.5%)	1.00	
	G/A	76 (35.7%)	84 (49.1%)	**1.81 (1.20–2.74)**	0.0048
	A/A	1 (0.5%)	4 (2.3%)	6.55 (0.72–59.64)	0.095
**Dominant**	G/G	136 (63.9%)	83 (48.5%)	1.00	0.0026
	G/A-A/A	77 (36.1%)	88 (51.5%)	**1.87 (1.24–2.82)**	
**Recessive**	G/G-G/A	212 (99.5%)	167 (97.7%)	1.00	0.1
	A/A	1 (0.5%)	4 (2.3%)	5.08 (0.56–45.81)	
**Over-dominant**	G/G-A/A	137 (64.3%)	87 (50.9%)	1.00	0.0079
	G/A	76 (35.7%)	84 (49.1%)	**1.74 (1.15–2.62)**	

### Distribution of allelic and genotypic frequency of C241T polymorphisms in *XRCC*3 gene

The frequency of **‘T’** allele was found to be predominant in UC group compared to controls (25% vs 19% respectively), with a 1.43 folds increased risk for UC (OR 1.43, 95% CI 1.01–2.01, *p* = 0.041) **(Table 3)**.

**Table 3:** *XRCC*3 genotypic distribution in patients with UC and healthy controls.

	Genotype	Controls	Patients	OR (95% CI)	*p*-value
**Co-dominant**	C/C	138 (64.8%)	89 (52%)	1.00	
	C/T	69 (32.4%)	78 (45.6%)	**1.75 (1.15–2.67)**	0.03
	T/T	6 (2.8%)	4 (2.3%)	1.03 (0.28–3.77)	0.95
**Dominant**	C/C	138 (64.8%)	89 (52%)	1.00	0.012
	C/T-T/T	75 (35.2%)	82 (48%)	**1.70 (1.12–2.56)**	
**Recessive**	C/C-C/T	207 (97.2%)	167 (97.7%)	1.00	0.77
	T/T	6 (2.8%)	4 (2.3%)	0.83 (0.23–2.98)	
**Over-dominant**	C/C-T/T	144 (67.6%)	93 (54.4%)	1.00	0.0081
	C/T	69 (32.4%)	78 (45.6%)	**1.75 (1.15–2.65)**	

CT genotype were found to be predominant in the UC group compared to controls (45.6%, 32.4% respectively, *p* = 0.03) with 1.75 folds increased risk for UC, which was statistically significant (OR 1.75, 95% CI 1.15–2.67, *p* = 0.03) **(Table 3)**. Based on the dominant model, combination of CT+TT genotypes were also observed are at high risk for UC (OR 1.70, 95% CI 1.12–2.56, *p* = 0.012) **(Table 3)**. Whereas in the recessive model, TT genotype (compared with CC+CT genotype) was insignificant in UC compare to control (OR 0.83, 95% CI 0.23–2.98, *p* = 0.77) but the overdominant model, CT genotype (compared with CC+TT genotype) is a risk genotype for UC (OR 1.75, 95% CI 1.15–2.65, *p* = 0.0082), further confirming the risk of **‘T’** allele in UC **(Table 3)**.

### Distribution of allelic and genotypic frequency of G135C polymorphisms in *RAD*51 gene

The *RAD*51 gene encodes protein involved in homologous recombinational repair (HRR) of double strand DNA (Cui *et al.*, 1999). The *RAD51* protein is responsible for the central activity of the HRR pathway, in which it catalyses the invasion of the broken ends of the DSB into the intact sister chromatid.

The **‘C’** allele frequency was found to be predominant in UC group (29%) compared to the control group (21%) **(Table 4)**.

**Table 4:** *RAD*51 genotypic distribution in patients with UC compared to healthy controls.

Model	Genotype	Controls	Patients	OR (95% CI)	*p*-value
**Co-dominant**	G/G	127 (59.6%)	78 (45.6%)	1.00	
	G/C	81 (38%)	86 (50.3%)	**1.73 (1.14–2.62)**	0.021
	C/C	5 (2.4%)	7 (4.1%)	2.28 (0.70–7.43)	0.171
**Dominant**	G/G	127 (59.6%)	78 (45.6%)	1.00	0.0062
	G/C-C/C	86 (40.4%)	93 (54.4%)	**1.76 (1.17–2.64)**	
**Recessive**	G/G-G/C	208 (97.7%)	164 (95.9%)	1.00	0.33
	C/C	5 (2.4%)	7 (4.1%)	1.78 (0.55–5.70)	
**Over-dominant**	G/G-C/C	132 (62%)	85 (49.7%)	1.00	0.016
	G/C	81 (38%)	86 (50.3%)	**1.65 (1.10–2.48)**	

GC genotypic frequency was found to be predominant in UC group (50.3%) compared to controls (38%) with the difference being statistically significant (*p* = 0.02). The GC genotype was found to be associated with 1.73 folds increased risk for UC compared to GG genotype (OR 1.73, 95% CI 1.14–2.62, *p* = 0.02) **(Table 4)**. Based on the dominant model, combination of GC+CC genotypes were also observed to be associated with high risk for UC (OR 1.76, 95% CI 1.17–2.64, *p* = 0.006). Based on the recessive model CC genotype did not show any statistical significant compare with the combination of GG+GC genotype (OR 1.78, 95% CI 0.55–5.70, *p* = 0.33) **(Table 4)**. Whereas overdominant model GC (Compared with GG+CC genotype) genotype were also observed to be associated with high risk for UC (OR 1.65, 95% CI 1.10–2.48, p = 0.016), further strengthening the association of **‘C’** allele with UC manifestation **(Table 4)**.

### Linkage Disequilibrium

In present study, pairwise LD estimates were obtained for the case and control group separately for the *hMSH*2, *XRCC*3 and *RAD*51 gene polymorphisms. The analysis revealed that most of the SNP marker combinations exhibited low LD scores, with the exception of few combinations that showed differential pattern of high LD scores in each of the analysis groups (cases and controls).

**Figure 1:**
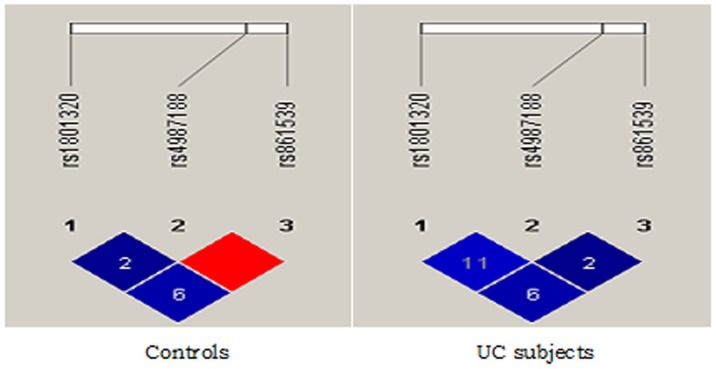
Linkage disequilibrium observed in control and ulcerative colitis groups.

While in the control group, the SNP loci combination of *hMSH*2: *XRCC*3 were in perfect LD. In contrast to this, the cases did not show any SNP loci combination with a perfect LD score **(Figure 1)**. Pairwise LD score were also calculated for the three polymorphisms studied **(Table 5)**.

**Table 5:** Pairwise Linkage Disequilibrium estimates in controls and Ulcerative colitis group.

	D′	LOD	r^2^
**Controls**			
*RAD*51: *hMSH*2	0.027	0.0	0.0
*RAD*51:*XRCC3*	0.065	0.14	0.004
*hMSH*2: *XRCC3*	1.0	1.67	0.053
**Ulcerative colitis**			
*RAD*51: *hMSH*2	**0.11**	**0.04**	**0.002**
*RAD*51:*XRCC3*	**0.068**	**0.09**	**0.004**
*hMSH*2: *XRCC3*	**0.021**	**0.01**	**0.0**

### Haplotype Analysis

Haplotype analysis is believed to be more informative approach in strengthening the genetic influence on disease manifestation than testing for individual genotypes, hence haplotypes were constructed based on the four polymorphisms and analyzed for the possible association with UC.

Of the all haplotypes obtained **(Table 6)**, one haplotypes carrying the recessive allele of *XRCC*3 and *RAD*51 polymorphism, G**TC** were found to be significantly predominant in the disease group. The G**TC** haplotype was found to be predominant in UC than controls with a **2.28** fold significant increase, (OR 2.28, 95% CI 1.08–4.83, *p* = 0.032). Another haplotypes carries the recessive allele of *hMSH*2 and *RAD*51 gene, **A**C**C** haplotype were found to be significant in UC when compared with control (OR 2.93, 95% CI 1.18–7.28, p = 0.021). Whereas the other haplotype did not shown any statistically significant. Hence these two haplotypes could be the risk haplotype for UC.

**Table 6:** Haplotype association with response.

Haplotype association with response (n = 384, crude analysis)								
	*hMSH*2	*XRCC*3	*RAD*51	Freq	Control	Patient	OR (95% CI)	P-value
1	G	C	G	0.4695	0.5006	0.43	1.00	—
2	A	C	G	0.1335	0.1462	0.1182	1.10 (0.56–2.18)	0.78
3	G	C	C	0.1246	0.1267	0.1242	1.42 (0.73–2.77)	0.31
4	G	T	G	0.1163	0.1396	0.0847	0.88 (0.45–1.72)	0.71
5	**G**	**T**	**C**	**0.0683**	**0.05**	**0.0921**	**2.28 (1.08–4.83)**	**0.032**
6	**A**	**C**	**C**	**0.055**	**0.0364**	**0.0761**	**2.93 (1.18–7.28)**	**0.021**

## Discussion

UC is a chronic inflammatory bowel disease (IBD) of unknown aetiology. The pathophysiology of UC relates to a dysregulated mucosal immune response to antigenic stimulation from gut microbiota on a background of genetic susceptibility [20].

Our findings elucidate that the GA genotypic frequency of *hMSH*2 was relatively higher in patients than in control (OR 1.81, 95% CI 1.20–2.74, *p* = 0.004) which is in absolute conformity with data of Poplawski *et al.*, (2006) [21] Significant association of *Gly*322*Asp* polymorphism of the *hMSH2* gene with breast cancer and colorectal cancer [22] was reported. The frequency of A allele was found to be predominant in UC group compared to controls, with a 1.64 folds increased risk for UC (OR 1.64, 95% CI 1.16–2.31, *p* = 0.004).

Our study on genotype CT of *XRCC3* gene polymorphisms showed statistical significance with an OR of 1.75 and 95% CI of 1.15–2.67, *p* = 0.03. Interestingly, similar result was observed in melanoma and bladder cancer [18], [12]. Improta *et al.*, (2008) [23] demonstrated a significant association between the *XRCC*3 Thr241Met polymorphism and colorectal and lung cancer. This *XRCC*3 codon 241 polymorphism was shown to have a significant association with colorectal [24] and lung [14] cancer risk; hence, our findings are in agreement with those reported by Mort *et al.*, (2003) [24]. But no association was found between this polymorphism and squamous cell carcinoma of the head and neck [6], gastric cancer [25] or basal cell carcinoma [26]. The heterozyte (CT) and homozygote variant (TT) genotypes were associated with a decreased risk of bladder cancer but these results were not significant [27]. The study of Shen *et al.* (2003) [28] was the first and the only one to suggest a protective role of *XRCC*3 codon 241 polymorphism against bladder cancer risk. The frequency of T allele was found to be high in UC group when compared to controls with a 1.43 folds increased risk for UC (OR 1.43, 95% CI 1.01–2.01, *p* = 0.041).

In *RAD*51, GC genotypic frequency was found to be predominant in UC group (50.3%) compared to controls (38%) with the difference being statistically significant (*p* = 0.02). We found that the SNP in the *RAD*51 gene GC genotype (OR 1.73, 95% CI 1.14–2.62, *p* = 0.02) may be an important predictive determinant for UC. Hosseini *et al.*, (2013) [29] also demonstrated that there was a significant association of breast cancer risk with *RAD*51 polymorphism. Wang *et al.*, (2001) [30] identified a *RAD*51 SNP that may be associated with an increased risk of breast cancer and a lower risk of ovarian cancer. Krupa *et al.*, (2011) [31] found that CC genotype decreased the risk of colorectal cancer in the Polish population. Other data has shown that GC heterozygote of the *RAD*51 polymorphism may be associated with the increased risk of colorectal cancer development [32]. Makowska *et al.*, (2012) [33] demonstrated that the variant genotypes of the CC RAD51 polymorphism may be positively associated with colorectal carcinoma in the Polish population. The homozygote variant (CC) genotypes was not associated in UC cohort (OR 2.28, 95% CI 0.70–7.43, *p* = 0.17). However C allele frequency was found to be high in UC group (29%) when compared to the control group (21%). GC genotypic frequency was found to be predominant in UC group (50.3%) when compared to controls (38%) with the difference being statistically significant (*p* = 0.02).

In the control group, the SNP loci combination of *XRCC*3:*hMSH*2 were in perfect LD, there was one other combination that demonstrated a moderate LD effect, i.e XRCC3: RAD51 (D′ = 60). In contrast to this, the cases did not show any SNP loci combination with a perfect LD score. In a recent report, it was found that the 399Gln allele of *XRCC*1 was in complete linkage disequilibrium (LD) with the 280His allele of same gene (D′ = 1.0) and the 280His allele was in complete LD with the 194Arg allele in *XRCC*1 gene (D′ = 1.0) in both whites and African Americans [21] in cutaneous melanoma. G**TC and ACC** haplotypes could be the risk haplotypes for UC. T′ and ‘C’ alleles of *XRCC*3 and *RAD*51 respectively contribution being significant in risk stratification of UC. Sant et al., (2011) [34] identified that the CTC haplotype in BRCA1 gene was significantly associated with decreased mean number of breaks per cell (MBPC).

Further studies on the epistatic interactions are warranted to elucidate their possible underlying mechanisms. Since different populations have distinct genetic backgrounds, it is necessary to validate or replicate such associations with independent samples collected from other ethnic groups/populations.

## Supporting Information

Table S1 **Haplotype data.**
(XLSX)
